# AI-Enabled Precision Nutrition in the ICU: A Narrative Review and Implementation Roadmap

**DOI:** 10.3390/nu18010110

**Published:** 2025-12-28

**Authors:** George Briassoulis, Efrossini Briassouli

**Affiliations:** 1 Postgraduate Program “Emergency and Intensive Care in Children, Adolescents and Young Adults”, School of Medicine, University of Crete, 71003 Heraklion, Greece; 2 Second Department of Paediatrics, Aglaia Kiriakou Children’s Hospital, School of Medicine, National and Kapodistrian University of Athens, 10679 Athens, Greece; efroelesar@hotmail.com

**Keywords:** artificial intelligence, critical care nutrition, precision nutrition, enteral and parenteral nutrition, reinforcement learning, clinical decision support

## Abstract

**Background:** Artificial intelligence (AI) is increasingly used in intensive care units (ICUs) to enable personalized care, real-time analytics, and decision support. Nutritional therapy—a major determinant of ICU outcomes—often remains delayed or non-individualized. **Objective:** This study aimed to review current and emerging AI applications in ICU nutrition, highlighting clinical potential, implementation barriers, and ethical considerations. **Methods:** A narrative review of English-language literature (January 2018–November 2025) searched in PubMed/MEDLINE, Scopus, and Web of Science, complemented by a pragmatic Google Scholar sweep and backward/forward citation tracking, was conducted. We focused on machine learning (ML), deep learning (DL), natural language processing (NLP), and reinforcement learning (RL) applications for energy/protein estimation, feeding tolerance prediction, complication prevention, and adaptive decision support in critical-care nutrition. **Results:** AI models can estimate energy/protein needs, optimize EN/PN initiation and composition, predict gastrointestinal (GI) intolerance and metabolic complications, and adapt therapy in real time. Reinforcement learning (RL) and multi-omics integration enable precision nutrition by leveraging longitudinal physiology and biomarker trajectories. Key barriers are data quality/standardization, interoperability, model interpretability, staff training, and governance (privacy, fairness, accountability). **Conclusions:** With high-quality data, robust oversight, and clinician education, AI can complement human expertise to deliver safer, more targeted ICU nutrition. Implementation should prioritize transparency, equity, and workflow integration.

## 1. Introduction

Artificial Intelligence (AI) is emerging as one of the most innovative tools in modern medicine, enabling the analysis of complex datasets, improving clinical decision-making, and supporting personalized therapeutic interventions—including nutritional therapy. AI is expected to bring significant change through the use of advanced machine learning models and large language models (LLMs), offering a broad spectrum of applications [1].

In Intensive Care Units (ICUs), where data volume and complexity are exceptionally high and diagnostic–therapeutic cycles are extremely short, AI demonstrates unique potential. Despite the rapid progress in this field, however, few AI-based solutions have yet been integrated into daily clinical practice, largely because of methodological and ethical challenges [2].

To date, AI applications in critical-care nutrition remain relatively limited, with most work focused on malnutrition screening and forecasting enteral-feeding tolerance. Although the available evidence is still insufficient to support broad implementation, AI holds considerable promise for individualized nutritional management. Its key advantage is the capacity to fuse multiple, heterogeneous data streams, capturing the rapidly evolving physiology of critical illness while accommodating substantial inter-patient variability [3]. Because the majority of published AI nutrition studies have been conducted in adult ICUs, this review primarily focuses on adult populations while explicitly highlighting pediatric ICU applications where evidence is available.

Importantly, the ethical and practical deployment of AI is now viewed as equally important as its technical development. Recent expert consensus has highlighted the need to align AI innovation with humanized, patient-centered care, so that progress toward personalized medicine preserves fairness, transparency, and clinician accountability. In this context, “human-centric AI” is emphasized as a tool that supports rather than supplants clinical judgment, reinforcing empathetic care and protecting the therapeutic relationship [4].

In contrast to previous reviews that have mainly provided technology-focused overviews of AI in critical care or addressed isolated tasks such as energy-expenditure prediction or malnutrition screening, this narrative review is explicitly organized along the ICU nutrition pathway (assessment → implementation → monitoring → adaptive control). We link specific AI techniques (ML, DL, NLP, RL) to clearly defined nutritional tasks, summarize the maturity of the evidence from conceptual modeling through external validation to early clinical implementation, and propose a pragmatic implementation roadmap that integrates ethical, governance, and workforce considerations. Our aim is therefore to offer an ICU nutrition-centric, implementation-oriented framework that complements existing generic AI-in-ICU reviews rather than duplicating them.

## 2. Materials and Methods

We conducted a narrative review to synthesize current and emerging applications of artificial intelligence (AI) for nutritional therapy in intensive care. Reporting was guided by SANRA criteria for narrative reviews [5]. AI-assisted language editing was used for grammar and syntax only, with all changes reviewed by the authors.

### 2.1. Data Sources and Search Strategy

PubMed/MEDLINE, Scopus, and Web of Science were searched for English-language literature from January 2018 to November 2025. A pragmatic Google Scholar sweep and backward/forward citation tracking complemented database searches. Core search strings combined AI-related terms (e.g., “artificial intelligence”, “machine learning”, “deep learning”, “neural network*”, “reinforcement learning”, “natural language processing”) with nutrition- and ICU-related concepts (e.g., “critical care”, “intensive care”, “ICU”, “enteral nutrition”, “parenteral nutrition”, “energy expenditure”, “indirect calorimetry”, “protein dosing”, “malnutrition”, “feeding tolerance”, “refeeding syndrome”, “metabolomics”, “multi-omics”). Search strings were adapted for each database and used Boolean operators and truncation where appropriate. Reference lists of key articles and relevant guidelines were also screened manually to identify additional studies.

### 2.2. Eligibility Criteria

We included publications that met all of the following criteria: (i) human studies in adult or pediatric ICU settings, or studies in closely related high-acuity environments (e.g., high-dependency or step-down units) judged to be directly transferable to ICU nutrition practice (similar hemodynamic instability, organ support, and nutrition-therapy questions); (ii) use of AI/ML methods (including machine learning, deep learning, natural language processing, or reinforcement learning) for at least one step of the nutrition pathway: assessment of nutritional risk or requirements, prescription (timing, route, composition), monitoring of delivery/tolerance, or prediction/prevention of nutrition-related complications; and (iii) designs with clear clinical relevance, including implementation or validation studies, large observational datasets, methodological papers with explicit ICU-nutrition tasks, and major guidelines or position statements integrating AI perspectives. We excluded non-ICU settings without clear transferability to critical-care nutrition, animal studies, case reports and small case series, conference abstracts, non-peer-reviewed preprints, and purely technical AI papers without a defined nutritional endpoint.

### 2.3. Selection and Data Charting

Titles and abstracts were screened independently by both authors for potential relevance, with disagreements resolved by discussion and, when needed, re-review of the full text. No automation tools were used for study selection. For each included article, we charted study design (observational, model-development, external validation, implementation/pilot trial), population (adult vs. pediatric), data sources, AI method (e.g., tree-based ensembles, neural networks, natural language processing pipelines, reinforcement learning frameworks), clinical task (e.g., energy or protein estimation, malnutrition screening, intolerance prediction, adaptive control), validation strategy (internal vs. external, temporal or geographic), performance metrics, interpretability approach, and degree of clinical implementation. A schematic of the selection process is provided in Supplementary Figure S1.

### 2.4. Synthesis and Appraisal

Findings were synthesized thematically across the ICU nutrition pathway (assessment → implementation → monitoring → adaptive control), with additional cross-cutting domains addressing ethics, governance, interoperability, and workforce training. Because the included studies were methodologically diverse, with heterogeneous AI methods and outcomes, a formal meta-analysis was not feasible. In appraising the evidence, we placed greater weight on studies with external validation, clinically interpretable outputs, and patient-relevant endpoints.

This review has several limitations. First, as a narrative (non-systematic) review, it remains vulnerable to selection and publication bias despite broad database coverage and citation tracking. Second, observational ICU nutrition datasets are heavily confounded by illness severity, treatment coupling (e.g., co-variation of nutrition dose with vasoactive or ventilatory support), and center-level practice differences; these factors complicate causal inference and may inflate apparent model performance. Third, high-dimensional AI models are particularly prone to overfitting and data leakage when small samples, repeated measurements, or temporally overlapping training and test sets are used; many published reports do not provide sufficient detail to fully exclude these risks. Finally, most available AI studies do not report sex- or gender-stratified performance or explicitly address gender-related biases, which we consider an important unresolved limitation. Future ICU-nutrition AI models should therefore increasingly incorporate causal-inference frameworks, rigorous internal and external validation, and fairness assessments to better distinguish association from clinically actionable, equitable effects.

## 3. The Role of Nutritional Therapy in Intensive Care Units

Nutritional support is a cornerstone of ICU care, essential for preventing complications, promoting recovery, and reducing mortality among critically ill patients. Despite its recognized importance, the optimal nutritional strategy remains controversial, and achieving a truly individualized approach represents a major clinical challenge. Current evidence-based guidelines for nutrition therapy in the critically ill—developed by the American Society for Parenteral and Enteral Nutrition (ASPEN) and the European Society for Clinical Nutrition and Metabolism (ESPEN)—provide comprehensive recommendations, yet their translation into bedside practice is often difficult, frequently resulting in either under- or over-feeding [6,7].

Both undernutrition and overnutrition are linked to increased morbidity and mortality, a higher risk of infectious and metabolic complications, and prolonged ICU stay. Accurate assessment of nutritional requirements and individualized delivery of nutrients are therefore critical for improving outcomes and prognosis. Traditionally, estimation of energy and protein needs has relied on predictive equations or on single-time indirect calorimetry measurements. However, these static methods fail to capture the dynamic metabolic fluctuations that accompany critical illness [8]. Consequently, while adequate nutrition is vital for maintaining physiological function, the rapidly changing metabolic demands of ICU patients make nutritional support a continuous and complex challenge [9].

### 3.1. Nutritional Support in ICUs: Challenges in Delivery and Monitoring

Nutritional support is a fundamental component of survival and recovery for critically ill patients in the ICU. Although strong evidence links adequate and balanced nutrition with reduced morbidity and mortality, the clinical implementation of nutrition therapy continues to face substantial weaknesses and delays [3].

#### 3.1.1. Early Enteral Nutrition and Individualized Advancement

Early initiation of EN within 24–48 h of ICU admission has generally been associated with improved outcomes, including fewer infectious complications and shorter duration of mechanical ventilation [6,7]. However, the benefit–risk balance is not uniform across hemodynamic phenotypes. In patients receiving vasopressor support, emerging evidence suggests that EN safety and effectiveness may depend on vasopressor dose and trajectory, as well as global perfusion adequacy, rather than on the binary presence of shock, supporting a more nuanced, individualized approach to EN timing and advancement [10].

Within this evolving evidence base, AI-enabled systems offer an opportunity to develop algorithms that estimate individual nutritional needs, adapt interventions in near real time, and improve monitoring of nutritional status [11]. By integrating continuous physiological, biochemical, and clinical data—including hemodynamic trajectories—such models may help clinicians optimize the timing, route, and composition of feeding more precisely than static, guideline-based approaches.

#### 3.1.2. Clinical Consequences of Underfeeding and Overfeeding

Undernutrition in ICU patients leads to impaired immune function, delayed wound healing, increased susceptibility to infection, and prolonged mechanical ventilation and ICU stay. Conversely, overfeeding—particularly excessive caloric or glucose intake—contributes to hyperglycemia, hypercapnia, and increased metabolic stress [12].

Incorporating AI-driven predictive mechanisms into clinical decision-support systems enables timely adjustment of feeding strategies and helps reduce complications such as gastrointestinal intolerance and fluid overload [13,14]. These tools may also enhance adherence to evidence-based feeding protocols and improve the precision of metabolic control.

### 3.2. Individualizing Route and Timing: Emerging Evidence for EN vs. PN

Although early EN has long been considered a core component of ICU care, evidence from landmark trials (e.g., CALORIES and NUTRI-REA-2) indicates that any advantage of EN over parenteral nutrition (PN) during the first week of critical illness is context-dependent, particularly in patients with shock or high vasopressor requirements. Accordingly, more recent guideline updates adopt a more individualized approach to route and timing, integrating clinical context, hemodynamic stability, and the risk of gastrointestinal intolerance when choosing EN versus PN in the acute phase [15].

This shift highlights the need for decision-support tools that better capture patient heterogeneity. In parallel, expert recommendations continue to emphasize uncertainty around optimal protein targets, noting that fixed high-protein prescriptions may not reflect the evolving metabolic phases of critical illness. AI-based systems are well suited to integrate real-time metabolic, clinical, and functional signals to manage this complexity and dynamically personalize nutrient delivery [16].

### 3.3. Estimating Nutritional Requirements: Limitations of Traditional Methods

Classical predictive equations—such as those of Harris–Benedict, Schofield, and Mifflin–St Jeor—are widely used to estimate basal energy expenditure, yet they often fail to reflect the rapidly changing metabolic demands of critically ill patients [17,18]. Although indirect calorimetry is considered the gold standard for determining resting energy expenditure, it is not always available and can be affected by technical or clinical limitations [19].

Recent studies have demonstrated the potential of AI-based algorithms to predict nutritional outcomes using parameters such as inflammatory status, energy balance, metabolic biomarkers, body composition, and illness severity [20]. Deep-learning models can accurately forecast the risk of developing malnutrition during the ICU stay [21].

Predictive modeling using AI thus opens new opportunities for dynamic monitoring of nutritional status and for identifying patients at high risk of under- or over-nutrition. Traditional screening tools, including the Nutritional Risk Screening 2002 (NRS-2002) and Subjective Global Assessment (SGA), suffer from subjectivity and limited reproducibility—especially in unstable, critically ill populations [22].

## 4. Artificial Intelligence: Basic Concepts and Technologies

Artificial Intelligence (AI) focuses on developing algorithms capable of performing tasks that traditionally require human intelligence—such as reasoning, learning, language comprehension, and pattern recognition. In medicine, diverse AI techniques are increasingly being applied to support diagnostic and therapeutic decision-making [23]. To avoid conceptual confusion, we use the following terminology in this review. A learning paradigm refers to the overarching strategy (e.g., supervised, unsupervised, or reinforcement learning). An algorithm family denotes a class of methods within a paradigm (e.g., gradient-boosted decision trees, random forests, convolutional neural networks, transformers). A model refers to a specific trained instance of an algorithm applied to a given dataset for a defined clinical task (for example, an XGBoost-based predictor of refeeding-syndrome hypophosphatemia). This hierarchy is used throughout the manuscript when describing AI applications in ICU nutrition.

### 4.1. Categories and Techniques of AI

AI encompasses several major subfields, the most relevant of which in clinical nutrition are summarized below.

Machine Learning (ML):

ML represents the most extensively used approach in AI-assisted nutrition therapy and includes three main types of learning paradigms:

Supervised Learning:

In this paradigm, algorithms are trained on labeled datasets to predict future outcomes or classify data. It has been applied to forecast the risk of malnutrition, intolerance to critical-care nutrition, or the likelihood of metabolic complications [24].

Unsupervised Learning:

Here, algorithms detect hidden patterns or clusters within unlabeled data, identifying sub-phenotypes or response profiles among critically ill patients that may correspond to different nutritional needs or tolerance levels [25].

Reinforcement Learning (RL):

RL algorithms learn by interacting with their environment, optimizing decisions through reward–penalty feedback loops. In ICU nutrition, RL can be used to continuously refine and personalize feeding strategies in real time according to a patient’s evolving physiological condition [26].

### 4.2. Neural Networks and Deep Learning (DL)

Artificial Neural Networks (ANNs) are computational models inspired by the human brain that excel at processing large and complex datasets [27].

Deep Learning (DL), a specialized subset of ML, utilizes multilayered neural networks to identify highly nonlinear patterns and generate advanced predictions. In the context of ICU nutrition, DL models can predict tolerance to critical-care nutrition, detect refeeding-syndrome risk, and estimate the likelihood of response to specific nutritional formulations [28].

### 4.3. Natural Language Processing (NLP)

Natural Language Processing (NLP) enables AI systems to interpret and analyze textual information from clinical narratives, such as progress notes, nutrition consults, and dietitian reports stored in electronic health records (EHRs). Recent work introduced the Framework for automatic Assessment of Nutritional Status (FANS), an NLP- and semi-supervised learning framework that extracts Global Leadership Initiative on Malnutrition (GLIM), SGA, and NRS-2002 malnutrition risk factors from free-text EHRs and classifies nutritional status, achieving high risk-factor identification performance (F1 ≈ 0.91) and good discrimination for malnutrition (AUROC ≈ 0.82) with SHAP-based interpretability [29]. In critical care nutrition, NLP can automatically extract information related to feeding tolerance, adverse events, medication–nutrition interactions, or historical dietary habits [30].

**Figure 1:**
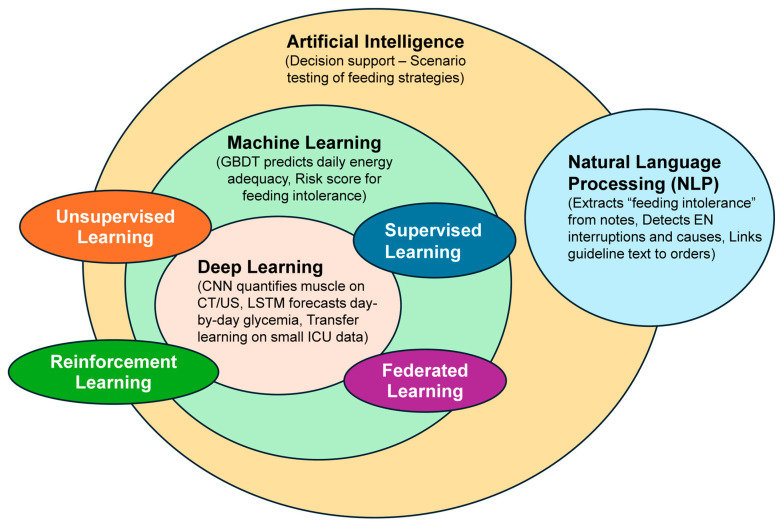
Conceptual map of AI domains relevant to ICU nutrition. ML/DL enable malnutrition risk prediction, energy/protein estimation, and adaptive optimization; NLP extracts nutrition-relevant signals from EHR narratives; federated learning supports multi-site model building while preserving privacy. Abbreviations: GBDT: Gradient Boosting Decision Trees; LSTM: Long Short-Term Memory; CNN: Convolutional Neural Network.

Integration of these technologies establishes the foundation for the next generation of “smart” nutrition-management systems in the ICU, aiming for fully personalized and adaptive interventions (Figure 1) [31].

## 5. Applications of AI in Nutritional Therapy in the ICU

The application of Artificial Intelligence in nutritional support for critically ill patients is expanding rapidly, with important advances across three main domains: nutritional assessment and prediction, implementation and monitoring of feeding therapy, and prevention of nutrition-related complications.

### 5.1. Assessment of Nutritional Requirements and Predictive Modeling

Recent advances highlight AI’s potential to enhance—and in some settings transform—traditional nutritional assessment. Computer-vision and machine learning approaches applied to 3D body scans, or even 2D smartphone images, show promise for accurate, contactless estimation of anthropometry and body composition, which could be particularly valuable for immobile ICU patients. These digital tools may enable more frequent and objective assessments while reducing clinician workload [32].

Accurate estimation of energy and protein requirements is essential yet often imprecise in critical care. AI-based systems offer opportunities to improve this process in several ways (Table 1).

### 5.2. Implementation and Monitoring of Nutritional Support

AI enables dynamic surveillance and real-time adaptation of feeding therapy through several interlinked systems:

Clinical Decision-Support Systems (CDSS):

These provide automated recommendations regarding formula composition, dosing, and modifications, based on constantly refreshed patient data [31].

Real-time adaptive feeding:

Reinforcement learning algorithms can fine-tune calorie and protein delivery continuously according to physiologic response [26].

Internet of Things (IoT) and wearable sensors:

Integration with feeding pumps or bedside databases allows continuous monitoring of intake, tolerance, and energy balance [37].

Smart infusion pumps with embedded AI:

These minimize human error and dynamically adjust infusion parameters to maintain prescribed targets [38].

### 5.3. Prediction of Nutrition-Related Complications

AI contributes to early detection and prevention of frequent nutrition-related adverse events:

Enteral feeding intolerance:

ML algorithms can identify patients unlikely to tolerate EN, enabling timely modification of strategy [39].

Refeeding-syndrome hypophosphatemia:

Predictive models such as XGBoost achieve over 90% accuracy in forecasting its occurrence, facilitating early preventive measures [40].

Aspiration, hypernatremia, and gastrointestinal intolerance:

Algorithmic analysis of feeding patterns, fluid balance, and serum sodium trajectories improves recognition and prevention of these complications [41].

Across these use-cases, most deployed models rely on supervised learning applied to structured ICU data, with tree-based ensemble methods (e.g., XGBoost, LightGBM, CatBoost) being particularly common because of their strong performance on tabular clinical variables and their compatibility with SHAP-based interpretability. Published ICU-nutrition models have primarily targeted process and intermediate outcomes—such as accurate classification of malnutrition risk, prediction of enteral-feeding intolerance, or refeeding-syndrome hypophosphatemia—rather than hard endpoints like mortality, infection rates, duration of mechanical ventilation, or long-term functional status. To date, the evidence base is dominated by retrospective model-development and internal-validation studies, with a smaller subset reporting external validation and only very few early implementation pilots [42]. Clearly distinguishing these maturity stages (conceptual models → predictive tools → embedded clinical decision support) is essential when interpreting performance metrics and when prioritizing candidates for prospective testing [43].

AI-driven predictive and monitoring systems therefore have the potential to shift ICU nutrition from static prescription to a responsive, precision-oriented process embedded within the broader digital critical-care ecosystem.

Moreover, AI applications are evolving from passive prediction to active, real-time optimization. Reinforcement learning (RL) approaches that integrate continuous physiological data to generate dynamic treatment recommendations are increasingly being explored in critical care [26]. Early-stage pediatric ICU initiatives applying RL to optimize sedation provide a useful conceptual framework that could be extended to nutrition support, where reward functions might balance delivery adequacy against signals of intolerance and metabolic complications [44].

## 6. Precision Nutrition Enabled by Artificial Intelligence

The implementation of Artificial Intelligence in individualized nutritional therapy closely aligns with the principles of precision medicine, which seeks to optimize care based on each patient’s unique characteristics. In critical illness, where metabolic responses vary markedly between individuals and across time, AI provides an unprecedented opportunity to achieve real-time personalization of nutritional support. Computational nutrition, an interdisciplinary field fusing AI (statistical modeling, simulation, causal inference, ML/DL), wearable biosensors, and multi-omics, aims to enable precision nutrition via prediction of personalized metabolic responses, individualized treatment-effect estimation, dynamic risk monitoring, and policy simulation [45]. Operationalizing these tools requires reliable sensors, bias monitoring, and interpretable models to maintain clinician trust and equitable benefit.

### 6.1. The Role of Reinforcement Learning (RL)

Reinforcement learning allows a computational system to adjust its actions through continuous interaction with the environment, using feedback in the form of rewards or penalties to refine decision strategies.

In ICU nutrition, RL algorithms can:

- *Adapt* caloric and protein dosing according to a patient’s evolving metabolic condition.
- *Analyze* dynamic changes in health status and feeding tolerance to fine-tune the nutrition plan in real time.
- *Integrate* into intelligent CDSS and ICU management platforms, facilitating continuous personalization of nutrition delivery [26,31,34].

Such adaptive systems may bridge the gap between static guideline recommendations and the fluid metabolic needs of critically ill patients.

### 6.2. Insights from Genomics and Metabolomics

Recent progress in genomics, epigenetics, and metabolomics enables identification of nutrient requirements and responses at the molecular level.

AI can combine genetic, biochemical, and physiological information to deepen understanding of what each patient specifically requires, enabling therapy tailored to individual metabolic phenotypes.

Emerging models further integrate gut-microbiome composition, immune signatures, and metabolic markers, forming the basis for multi-omic, highly personalized nutrition management in the ICU [46].

In parallel with omics-based approaches, several conventional and composite biomarkers have been proposed to refine precision nutrition in the critically ill. Examples include transthyretin (prealbumin), ratios that adjust trace elements for developmental stage (such as the selenium-for-age ratio), and lipid indices such as the LDL-to-total cholesterol ratio, which may reflect inflammation-related redistribution and endogenous substrate utilization in prolonged critical illness. Recent work suggests that AI models can integrate such markers alongside clinical and metabolic variables to improve discrimination of high-risk phenotypes, generate individualized protein and micronutrient targets, and monitor response to nutrition therapy over time [47]. Incorporating both routinely measured biomarkers and emerging omics-derived features is likely to be crucial for operationalizing truly molecularly informed precision nutrition at the bedside [48].

### 6.3. Identification of Nutritional Sub-Phenotypes in Critical Illness

ICU populations are heterogeneous, with widely varying metabolic and inflammatory profiles. AI methods can:

- *Detect subgroups* of patients characterized by distinct nutritional responses—such as high-inflammation or metabolic-suppression phenotypes.
- *Guide the choice* between enteral and parenteral nutrition, as well as the optimal formulation of energy, protein, fiber, and electrolyte content [49].
- *Support selection* of safer, more effective nutrition strategies for high-risk patients [50].

By enabling stratification and adaptive interventions, AI-driven precision nutrition moves beyond generalized guideline targets toward truly individualized, physiology-based therapy, improving efficacy and reducing complications.

By enabling data-driven stratification and adaptive interventions, AI-supported precision nutrition can move beyond generalized guideline targets toward individualized, physiology-informed therapy, with the potential to improve effectiveness while reducing adverse events.

Importantly, translating AI-enabled precision nutrition to the pediatric ICU requires explicit consideration of growth and neurodevelopment, age-dependent body composition, and rapidly changing substrate utilization across infancy, childhood, and adolescence. Pediatric datasets are typically smaller and more diverse, increasing the risk of overfitting and uneven performance, particularly across rare disease phenotypes. Integrating indirect calorimetry, VCO_2_-derived estimates, and longitudinal functional outcomes may be especially valuable in children, where the goals extend beyond survival to preserving developmental trajectories and long-term quality of life [8,17,18,35].

## 7. Prevention of Medical Errors and Improvement of Patient Safety

Artificial Intelligence not only supports individualized nutrition therapy but also plays a pivotal role in preventing medical errors, reducing adverse events, and enhancing overall patient safety in the ICU environment.

### 7.1. Prevention of Medication-Related Errors

Nearly half of medical errors in ICUs are medication-related, many of which directly or indirectly affect nutritional therapy.

AI-enabled systems can detect administration errors, cross-verify components, and alert for drug–nutrition incompatibilities (Table 2).

These capabilities substantially reduce the risk of inadvertent dosing errors and enhance the accuracy of parenteral-nutrition compounding and delivery.

### 7.2. Reduction of Alarm Fatigue

False or redundant alarms are a major contributor to clinician fatigue, distraction, and delayed responses in ICUs [52].

AI algorithms can:

- *Filter and prioritize alerts* according to clinical significance, reducing non-critical alarm repetition and system “noise.”
- *Evaluate the severity and context* of each event, enabling adaptive prioritization of interventions [53,54].

Through intelligent signal processing and contextual weighting, AI contributes to a calmer, safer ICU environment and more effective clinical workflow.

### 7.3. Workflow Optimization and Process Automation

AI further improves ICU efficiency by automating routine nutritional tasks, such as:

- *Recording and monitoring* nutrient intake and fluid balance.
- *Automatically recalculating* caloric and protein targets based on updated metabolic data.
- *Synchronizing with EHR systems* to update feeding plans and compliance reports.

Such automation enhances multidisciplinary collaboration, decreases manual data entry, and promotes adherence to evidence-based nutrition guidelines [34,52,53]. By optimizing workflow, AI allows clinicians to focus on higher-value clinical decisions while maintaining a high level of safety and consistency in nutritional management.

## 8. Challenges to the Implementation of AI in Nutritional Therapy

Despite AI’s promising capabilities, large-scale adoption in clinical nutrition—particularly in ICUs—remains limited. Key barriers can be grouped into three domains: data quality, economic feasibility, and ethical–legal considerations.

A major obstacle to real-world deployment of AI-enabled precision nutrition is the lack of standardized, high-granularity nutrition data across EHRs and bedside systems. Beyond prescribed targets, robust models require structured capture of delivered nutrition, including EN/PN composition; rate adjustments; interruptions and their documented reasons (procedures, intolerance, hemodynamic instability); use of prokinetics; gastric-residual protocols where applicable; stool output/diarrhea markers; the glucose variability—glycemic-management context; and nutrition-relevant losses during RRT. Linking feeding-pump logs and harmonizing these elements into a hospital-level “nutrition data dictionary” could markedly improve data completeness, external validation, and portability of AI tools. Establishing minimum datasets and interoperable definitions is therefore a prerequisite for safely scaling adaptive decision support in ICU nutrition.

### 8.1. Data Availability and Quality

AI performance and reliability hinge on the quality, completeness, and representativeness of the data used for training and validation; the main challenges are summarized in Table 3.

To mitigate these limitations, emerging approaches such as federated learning enable decentralized model training across multiple institutions without transferring patient-level data, thereby supporting privacy-preserving collaboration and improving generalizability while remaining compliant with data-protection regulations [60]. However, federated learning does not obviate the need for high-quality, standardized inputs: institutions must still invest in data infrastructure and specialized personnel to curate, harmonize, and maintain nutrition-relevant variables and labels. Persistent under-representation of older adults, women, patients with obesity, and rare-disease phenotypes further highlights the need for sex- and gender-aware performance reporting and fairness audits. Collectively, these data gaps translate into higher implementation costs and can slow adoption, particularly in resource-limited centers.

### 8.2. Economic and Infrastructural Challenges

The development, deployment, and maintenance of AI systems entail substantial financial and logistical demands (Table 4).

Achieving cost-effectiveness will require strategic investment, shared data frameworks, and demonstration of tangible clinical benefits that justify expenditure.

### 8.3. Ethical and Legal Considerations

The integration of AI into healthcare raises complex ethical and regulatory questions that must be addressed before widespread adoption (Table 5).

The emergence of Explainable Artificial Intelligence (XAI) seeks to reconcile algorithmic performance with interpretability, supporting ethical, fair, and clinically trustworthy systems [24,57,58,59]. Accordingly, institutions should maintain clear documentation (e.g., model provenance, versioning, and intended use) and enforce human-oversight checkpoints for high-impact recommendations. Beyond transparency and bias, an increasingly important challenge is defining a “social contract” for AI in healthcare, clarifying the roles, responsibilities, and accountability of clinicians, developers, regulators, and patients so that deployment aligns with core medical values and demonstrates patient benefit. Recent guidance therefore recommends establishing dedicated, multidisciplinary AI oversight committees to govern implementation, auditing, and risk mitigation [68]. In parallel, the concept of human–AI synergy emphasizes designing systems for combined performance that exceeds what either clinicians or algorithms achieve alone, positioning AI as an augmentative tool embedded within the ICU team rather than a replacement for clinical judgment [2].

Operationalizing these principles in ICU nutrition requires an explicit model-lifecycle framework. Before clinical activation, systems should undergo silent-mode evaluation to benchmark performance against standard care, followed by staged implementation with predefined escalation pathways and human-override rules for high-risk scenarios. After deployment, continuous monitoring for calibration drift, periodic re-validation as case-mix and practice evolve, and auditable links between recommendations and outcomes are essential. Embedding these safeguards within multidisciplinary governance structures helps ensure that adaptive nutrition models remain clinically reliable, equitable, and aligned with guideline-based care. Addressing these ethical, legal, and lifecycle requirements is necessary to move AI from proof-of-concept toward trustworthy, scalable bedside decision support in ICU nutrition.

## 9. Education of Healthcare Professionals and Organizational Culture

The successful integration of Artificial Intelligence into ICU nutritional therapy depends not only on technological sophistication but also on the human dimension—the education, acceptance, and collaboration of healthcare professionals. Without adequate understanding and engagement, even the most advanced AI tools may fail to achieve clinical impact.

### 9.1. Need for AI Literacy Among Healthcare Professionals

Physicians, dietitians, pharmacists, and nurses must develop a working understanding of how AI algorithms operate, their strengths and limitations, and when their recommendations can be safely trusted [66].

Educational initiatives should include:

- Technical skills for effective use of AI-based software and digital tools.
- Critical appraisal of AI outputs to differentiate between reliable and potentially biased recommendations [69,70].
- Training in data ethics, confidentiality, and responsible data management [71,72].

This education should be introduced early—within undergraduate and postgraduate curricula—and reinforced through lifelong learning and specialized professional programs [70,71]. Building “AI literacy” will empower clinicians to act as informed users and partners rather than passive recipients of algorithmic suggestions. Nutrition-specific AI literacy should cover protein/energy over- or under-prescription risks, EN intolerance signals, and recognition of spurious correlations in small datasets.

### 9.2. Managing Change and Promoting Acceptance Among Staff

Technological innovation in healthcare often meets resistance due to uncertainty, lack of familiarity, or fear of role displacement [36,69]. Common concerns include:

- *Fear* that AI may replace human clinical judgment.
- *Difficulty* adapting to new digital workflows.
- *Doubts* about the validity and transparency of algorithmic outputs [73,74].

To overcome these barriers, healthcare organizations must foster transparency, engage staff actively in implementation processes, and provide continuous technical and ethical support. Early involvement of end-users in AI deployment increases trust, usability, and sustainability [75].

### 9.3. Fostering Interdisciplinary Collaboration and Integration into Clinical Practice

Successful implementation of AI in ICU nutrition requires multidisciplinary collaboration among:

- Physicians and intensivists;
- Clinical dietitians and nutrition scientists;
- Nurses and pharmacists;
- Computer scientists and data engineers;
- Bioethicists and legal experts [45,50].

Co-designing solutions from the earliest stages—based on real clinical needs—enhances functionality and long-term adoption [34,38,76].

Furthermore, cultivating an organizational culture of innovation, where education, feedback, and continuous evaluation are encouraged, is essential for effective integration of AI into clinical workflows [75]. Such a culture transforms technology from an external tool into a trusted partner within the ICU team (Box 1).

Box 1 **Practical clinical use-case: AI-enabled precision nutrition in the ICU.**

    A mechanically ventilated patient is admitted with septic shock. During the first 24 h, an AI-enabled nutrition module continuously extracts and integrates real-time data from the EHR and bedside devices, including vasopressor dose and trajectory, lactate kinetics, fluid balance, renal function and RRT status, glycemic variability, inflammatory markers, and (when available) ventilator-integrated indirect calorimetry for resting energy expenditure (REE).

    In the early unstable phase, the system recommends a cautious, stepwise enteral nutrition (EN) approach and explicitly communicates uncertainty, providing an interpretable rationale (e.g., escalating vasopressor requirements with rising lactate suggesting impaired perfusion). As hemodynamics stabilize over the next 48 h, recommendations adapt toward early hypocaloric EN (e.g., <70% of measured REE), followed by progressive advancement toward REE while continuously estimating intolerance risk. Targeted alerts highlight modifiable contributors to intolerance (e.g., sedation depth, opioid burden, electrolyte disturbances) and support bedside troubleshooting.

    If intolerance persists or ischemic-risk indicators increase, the model suggests temporary de-escalation, adjustments in formula characteristics, or—after the first ICU week—an EN–PN hybrid strategy when appropriate. During recovery, energy and protein targets are recalibrated in line with current ESPEN/ASPEN guidance and functional goals, integrating rehabilitation intensity and renal/hepatic tolerance signals. Overall, this use-case illustrates how AI can support phase-aware route selection, dosing, and complication prevention, while preserving clinician oversight through transparent explanations and uncertainty-aware outputs.

## 10. Future Directions and Research Perspectives

Artificial Intelligence in critical-care nutrition is still in its infancy. Nevertheless, the field is evolving rapidly and presents broad research and clinical opportunities for developing predictive, adaptive, and precision-based nutrition models.

### 10.1. Timing and Composition of Nutritional Intervention

AI may help determine the optimal timing and route for initiating enteral (EN) or parenteral nutrition (PN) by moving beyond one-size-fits-all strategies [10,77]. By analyzing large, longitudinal datasets that incorporate hemodynamic trajectories, vasopressor dose trends, gastrointestinal tolerance markers, and evolving organ function, algorithms could identify which patients are likely to benefit from early EN and which may require delayed, stepwise initiation or primary PN during the acute phase of illness [78,79]. Such stratified decision support is particularly relevant in high-risk phenotypes in whom the margin between nutritional benefit and intolerance or ischemic risk is narrow.

Machine-learning models may also support optimization of macronutrient and micronutrient composition and the rate of feeding advancement, potentially reducing intolerance and metabolic instability. Because metabolic capacity and protein tolerance vary across acute, recovery, and rehabilitation phases, fixed high-protein targets may not consistently align with physiologic readiness. Accordingly, phenotype- and phase-specific protein dosing—integrating signals such as organ function, nitrogen balance/urea generation, urea/creatinine ratio, inflammatory activity, and rehabilitation intensity—may better match metabolic capacity, maximize functional recovery, and limit iatrogenic nitrogen burden [16,80]. This approach is intended to complement, not replace, guideline targets by dynamically refining bedside delivery as patients transition from catabolism and a hypometabolic state to recovery and a hypermetabolic state.

### 10.2. Selection and Formulation of Nutritional Products

For patients with chronic, complex, or disease-specific needs, EN formula choice (osmolality, caloric density, protein, fiber, micronutrients) is still driven mainly by expert preference rather than data [81]. AI-enabled systems track metabolic responses in real time and suggest tailored changes to nutrient mix and infusion regimen [40], aiming to enhance tolerance, limit gastrointestinal events, and accelerate recovery.

### 10.3. Prediction and Prevention of Complications

Advanced AI algorithms enable early detection of metabolic and gastrointestinal complications, including aspiration, dysglycemia, and feeding intolerance [16,79].

By recognizing complex interaction patterns among vital signs, laboratory results, and feeding data, these systems can anticipate deteriorations and prompt preventive action [41].

### 10.4. Dynamic Personalization Through Reinforcement Learning

Reinforcement learning (RL) systems can adjust nutrition delivery in real time by selecting enteral versus parenteral nutrition, tailoring dosing and frequency, and managing feeding interruptions or stepwise restarts based on evolving physiologic and biochemical signals [26]. By learning from longitudinal ICU trajectories, RL may support bedside decisions that are context-aware and aligned with dynamic metabolic tolerance, thereby bridging static guideline targets and the rapidly changing nutritional needs of critically ill patients.

A complementary future direction is the use of AI to address persistent limitations in critical-care nutrition research. Randomized nutrition trials have often struggled to demonstrate consistent benefits, in part because population heterogeneity can dilute treatment effects. Machine-learning methods may improve trial design through patient enrichment, identifying subgroups most likely to benefit (or be harmed) by specific nutritional strategies and thereby improving signal detection and reducing contradictory results [82]. This approach aligns with precision-nutrition goals and may accelerate the development of phenotype- and phase-specific feeding protocols supported by higher-quality evidence.

Together, RL-driven adaptive bedside decision support and AI-enabled trial enrichment provide a coherent pathway toward clinically meaningful, evidence-based personalization of ICU nutrition.

### 10.5. Integration of Novel Biomarkers and Multi-Omics Data

Combining genomics, metabolomics, proteomics, and microbiome data within AI frameworks promises to:

- *enable* more targeted, molecular-level nutrition strategies,
- *identify* distinct nutritional phenotypes, and
- *support* personalized pharmaco-nutrition approaches [83].

Such integration moves ICU nutrition beyond population-based guidelines toward biologically individualized care.

Recent work used serial untargeted metabolomics across the first ICU week to profile acute-phase energy metabolism, annotating >100 metabolites and identifying a prominent arginine-biosynthesis signal with increasing ornithine and L-arginine. Metabolic trajectories correlated with organ dysfunction (SOFA), suggesting feasibility for phase-specific nutritional tailoring and AI feature generation from temporal omics data [66].

### 10.6. Current Outlook

AI currently demonstrates the potential to:

- *Optimize* estimation of caloric and protein needs;
- Enhance individualization of nutrition therapy;
- Predict complications early;
- *Recommend* appropriate interventions in real time [2].

However, full realization of this potential requires:

- *Access to high-quality*, standardized datasets;
- Improved interoperability among EHR systems;
- Dedicated training of healthcare professionals;
- *A robust ethical and regulatory framework* safeguarding patient autonomy and data privacy.

At present, therefore, AI has mainly improved risk stratification and process measures in ICU nutrition, whereas consistent evidence of benefit on hard clinical outcomes such as mortality, major infections, or ventilator-free days remains limited and represents a key research gap.

AI should thus be viewed as a complementary tool, not a substitute for clinical judgment. The goal is a human-centered, technology-enhanced care model that ensures safety, efficiency, and equity in ICU nutritional support [84].

**Figure 2:**
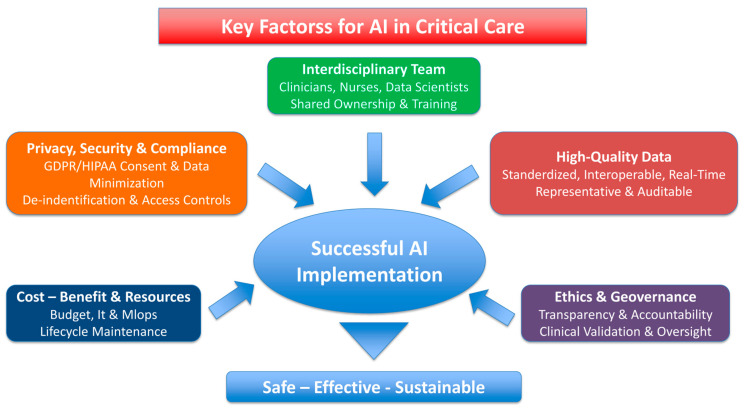
Current and future AI applications across the ICU nutrition pathway: assessment (needs and risk), implementation (timing, route, composition), monitoring (tolerance, complications), and adaptive control (RL). The shading also reflects the maturity of evidence from conceptual models to clinical implementation.

Collaboration among clinicians, data scientists, technologists, patients, and regulatory bodies will be the key to sustainable and ethically responsible integration of AI into future critical-care nutrition (Figure 2).

### 10.7. Future Research Priorities

Upcoming research should focus on:

- *Comparative evaluation* of AI-based versus traditional nutrition-assessment methods;
- Multicenter validation and standardization of predictive models;
- *Ethical and regulatory integration* of AI systems into clinical practice frameworks.

Such studies will define evidence-based pathways for safe, transparent, and clinically meaningful AI deployment in nutritional therapy.

## 11. Conclusions

Nutritional therapy in the Intensive Care Unit remains a cornerstone of critical care, directly influencing survival, complication rates, and recovery among critically ill patients. The integration of Artificial Intelligence (AI) into daily clinical nutrition practice has the potential to fundamentally transform the management of feeding in critical illness.

AI technologies can support every step of nutritional care—from early risk assessment and prediction of energy and protein needs to dynamic adaptation of feeding strategies and prevention of complications. Through machine learning, deep learning, and reinforcement learning algorithms, AI enables real-time, data-driven personalization that complements clinical judgment and promotes safer, more effective therapy.

Nevertheless, meaningful implementation requires high-quality data, interdisciplinary collaboration, transparent and explainable models, and the education of healthcare professionals. The ethical use of AI must preserve patient autonomy and trust while ensuring accountability and fairness.

When appropriately designed and responsibly integrated, AI serves not as a replacement but as a partner to human expertise, fostering a new era of precision nutrition in critical care—one that combines technological innovation with compassionate, individualized patient management.

## Figures and Tables

**Table 1:** Artificial intelligence approaches for individualized energy and protein targets in the ICU.

Developing	Individualized predictive models using ML and DL techniques that combine data from vital signs, laboratory results, inflammatory biomarkers, and indicators of physical activity [31,33].
Improving	The prediction of nutritional requirements when integrated with indirect calorimetry, surpassing static equations such as Harris–Benedict [34,35].
Utilizing	AI-supported screening tools, such as MUST-Plus, which draw upon EHR data and machine learning classifiers to identify patients at risk of malnutrition with greater accuracy and sensitivity compared with traditional scores (e.g., MUST, NRS-2002) [20].
Generating	Algorithms to predict complications—such as enteral feeding intolerance or hypophosphatemia—by analyzing large, continuously updated datasets collected during ICU stay [36].

**Table 2:** AI-enabled prevention of medication- and nutrition-related administration errors.

Error detection	Detect, in real time, errors in drug or nutrient solution administration (incorrect dosage, infusion rate, or incompatibility).
Cross-verification	Utilize computer vision and barcode-based verification to identify and cross-check infusion components before delivery.
Incompatibility alerts	Flag potential drug–nutrition incompatibilities, thereby improving pharmaco-nutritional safety [51].

**Table 3:** Data availability and quality considerations for AI performance.

Nutritional databases	Specialized, high-resolution databases focused on ICU nutritional variables remain scarce [55].
Existing datasets	Resources such as MIMIC provide value but are often heterogeneous, have substantial missingness, and lack nutrition-specific annotation/labels for modeling [56].
Bias in data	Gender-related biases, and under-representation of older adults, patients with obesity, and rare-disease populations can introduce bias, reducing prediction accuracy and equity across subgroups [24,54,55,57,58,59].

**Table 4:** Economic and infrastructural challenges of AI systems.

Infrastructure	High-performance computing infrastructure, ongoing software maintenance/updates, and specialized technical expertise are required [61].
Cost	Despite falling prices for cloud services and GPU processing, total costs remain prohibitive for many healthcare institutions [62].
Funding	Most hospitals lack sustainable, long-term funding mechanisms to maintain, monitor, and update AI-based decision-support systems [63].

**Table 5:** Ethical and legal considerations regarding the integration of AI into healthcare.

Privacy and GDPR compliance	Health data are highly sensitive and require robust de-identification/anonymization, role-based access controls, consent management, and auditable logs [23,64].
Transparency and explainability	Many high-performing models operate as “black boxes”; limited interpretability can undermine clinician and patient trust and uptake [12,64,65].
Responsibility and accountability	When AI-influenced decisions lead to adverse outcomes, delineation of professional liability and system accountability remains unclear; clear governance and documentation are needed [58,66,67].

## Data Availability

No new data were created or analyzed in this study.

## Supplementary Materials

The following supporting information can be downloaded at: https://www.mdpi.com/article/10.3390/nu18010110/s1. Supplementary Figure S1: Flow diagram of the narrative selection process.

## Abbreviations

AI	Artificial Intelligence
ICU	Intensive Care Unit
EN	Enteral Nutrition
PN	Parenteral Nutrition
REE	Resting Energy Expenditure
IC	Indirect Calorimetry
ML	Machine Learning
DL	Deep Learning
RL	Reinforcement Learning
NLP	Natural Language Processing
XAI	Explainable AI
EHR	Electronic Health Record
GLIM	Global Leadership Initiative on Malnutrition
SGA	Subjective Global Assessment
NRS-2002	Nutritional Risk Screening 2002
SOFA	Sequential Organ Failure Assessment
GDPR	General Data Protection Regulation
HIPAA	Health Insurance Portability and Accountability Act
