# Supplementary figures and images for: AI-Enabled Precision Nutrition in the ICU: A Narrative Review and Implementation Roadmap
Source: Nutrients. 2025 Dec 28;18(1):110. doi: 10.3390/nu18010110 (PMC12787373; doi:10.3390/nu18010110)

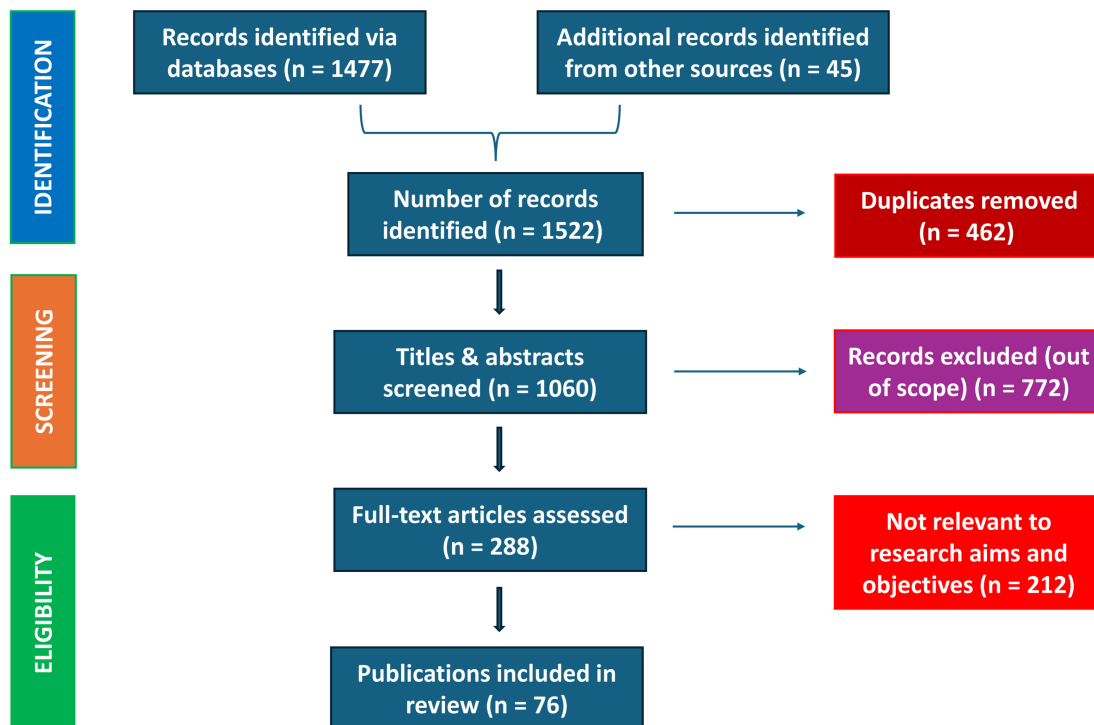

Supplementary Figure S1. Flow diagram of the narrative selection process.

Supplement: Supplementary file 1 [file nutrients-18-00110-s001.zip › nutrients-4063799-supplementary.pdf]
